# Overexpression of small nucleolar RNA SNORD1C is associated with unfavorable outcome in colorectal cancer

**DOI:** 10.1080/21655979.2021.1990194

**Published:** 2021-10-26

**Authors:** Yonghui Liu, Chengwen Zhao, Jing Sun, Guihua Wang, Shaoqing Ju, Chen Qian, Xudong Wang

**Affiliations:** a Department of Laboratory Medicine, Affiliated Hospital of Nantong University, Nantong, China; b The Faculty of Laboratory Medicine School of Public Health, Nantong University, Nantong, China

**Keywords:** Small nucleolar RNAs, SNORD1C, colorectal cancer, biomarker, diagnosis

## Abstract

Colorectal cancer (CRC) is the second most incident cancer and third leading cause of cancer-related mortality worldwide. Small nucleolar RNAs (snoRNAs) are small non-coding RNAs located in the nucleoli of cells, and play key roles in multiple cancers. However, the role of serum snoRNAs in CRC remains unknown. We analyzed the expression of the snoRNA SNORD1C in the serum of patients with CRC using quantitative real-time polymerase chain reaction (qRT-PCR) (n = 122). The receiver operating characteristic (ROC) curves were estimated, and the area under the ROC curve (AUC) was calculated. Gene ontology (GO) and Kyoto encyclopedia of genes and genomes (KEGG) analysis of co-expressed genes was performed using the database for annotation, visualization, and integrated discovery (DAVID), and visualized by R language. The results showed that the expression of SNORD1C in patients with CRC (n = 122) was significantly higher than that in normal individuals (n = 50) and patients with benign colorectal disease (n = 33) (*P* < 0.05). The overexpression of serum SNORD1C was related to poor tissue differentiation and high carcinoembryonic antigen (CEA) levels (*P* < 0.05). In the ROC curve analysis, SNORD1C serum expression combined with CEA offered better predictive value for the diagnosis of CRC (AUC = 0.838) compared with SNORD1C (AUC = 0.748) or CEA (AUC = 0.715) alone. High expression of SNORD1C was found to be closely associated with prognosis and unfavorable outcomes in patient with CRC. Therefore, serum SNORD1C may be a noninvasive tumor biomarker for diagnosis of CRC.

## 1. Introduction

Colorectal cancer (CRC) has become the primary cause of cancer-related deaths of the digestive system [1] and ranked third among the new cancer cases and cancer-related deaths in 2020 [2]. Although advances in medical technology have led to the popularization of cancer screening tests and the implementation of individualized medical programs, the prognosis of patients with CRC remains unsatisfactory [3,4]. Tumor recurrence, metastasis, and poor response to drug treatment are the main obstacles in the successful treatment and increase in survival time of patients. Therefore, exploring novel biomarkers for risk stratification and clinical management of patients with CRC has important implications.

Small nucleolar RNAs (snoRNAs) are a group of non-coding RNAs (ncRNAs) approximately 60–300 nucleotides in length. Based on their structural features, snoRNAs are classified into two main subtypes: C/D box snoRNAs and H/ACA box snoRNAs [5], which are thought to play a critical role in the modification, maturation, and stabilization of ribosomal RNAs (rRNAs) via the formation of small nucleolar ribonucleoproteins (SNORNPs) [6].

Mounting evidence supports the role of snoRNAs in cancers and growing number of snoRNAs with potential as biomarkers for early diagnosis and prognosis of cancers have been reported. Overexpression of SNORA23 in pancreatic ductal adenocarcinoma contributes to the growth and metastasis of xenograft tumors in mice [7]. Increased expression of SNORA21 in tissue correlates with advanced disease stage and worse patient outcome, and may therefore be used as a diagnostic and prognostic marker for CRC [8]. In addition, upregulated SNORD52 drives tumorigenesis via the Upf1/SNORD52/CDK1 pathway and may serve as a biomarker to predict poor prognosis for hepatocellular carcinoma [9].

Abnormal snoRNAs can be detected in serum and have diagnostic value. SNORA55 is detectable in serum in prostate cancer, and is consistent with the results of tissue detection, confirming the potential of SNORA55 as a new biomarker [10]. Although snoRNAs play important roles in the development of cancers, the diagnostic value of snoRNAs in the serum of CRC patients remains unclear. Mannoor et al. found that SNORD1C was expressed at higher levels in non-small cell lung cancer cells that were ALDH1+ compared to those that were ALDH1- [11]. ALDH1+ tumor cells are believed to have higher proliferation rates, self-renewal capacity, and tumorigenic potential *in vivo*, which constitute important causes of cancer recurrence [12,13]. Therefore, SNORD1C may have a driving effect on non-small cell lung cancer. However, the significance of SNORD1C in CRC has not yet been reported.

Herein, we hypothesized that SNORD1C may play a significant role in CRC. To study the role and clinical significance of SNORD1C in the serum of patients with CRC, we analyzed SNORD1C expression in serum samples from patients with CRC by qRT-PCR and evaluated its clinical implication in combination with CEA in blood. In addition, we performed Gene Ontology (GO) and Kyoto Encyclopedia of Gene and Genome (KEGG) analysis on SNORD1C co-expressed genes in the TCGA database. Taken together, our results suggest that SNORD1C may be a novel, feasible, and readily assessable biomarker for the diagnosis of CRC.

## 2 Materials and methods

### 2.1 Patients and serum samples

We collected specimens from the clinical laboratory of the Affiliated Hospital of Nantong University (Nantong, China) from March 2018 to June 2019. Serum was collected from 122 patients who were diagnosed with CRC by pathologists and did not receive any treatment, and of these, 50 samples were paired postoperative serum samples. In addition, serum was collected from 33 patients with benign colorectal diseases, including one patient with ulcerative colitis, one with Crohn disease, two with common enteritis, and twenty-nine with intestinal polyps. At the same time, serum was collected from 50 healthy individuals as a normal control group. Serum samples were collected following centrifugation of blood at 3000 ×g for 10 min and stored in RNase-free Eppendorf (EP) tubes at −80°C until they were used to extract total RNA.

### 2.2 RNA extraction, reverse transcription, and qRT-PCR

Total RNA was extracted from the sample using the TRIzol™ LS (Invitrogen Life Technology, Carlsbad, CA, USA) reagent, and each sample was diluted with 30 μL of nuclease-free water. Nanodrop method was used to measure the concentration of total RNA and to evaluate its quality. Samples that met the requirements for the reverse transcription reaction were selected and the RevertAid First Strand cDNA Synthesis Kit (K1622; Thermo Scientific, USA) was used to obtain complementary DNA (cDNA) from the total RNA. All RNA and cDNA samples were stored at −80°C until further analysis. The relative expression level of SNORD1C was determined using a Mastercycler nexus PCR instrument (Eppendorf, Germany) under the following conditions: One cycle at 95°C for 10 s (pre-denaturation), followed by 40 cycles at 95°C for 10 s, 60°C for 30 s, and 70°C for 30 s. The expression of SNORD1C in each sample was normalized to that of the internal control small nuclear RNA (U6). The relative expression level of SNORD1C was calculated using the 2^−ΔΔCt^ method, ΔΔCt = mean of the experimental group (Ct_SNORD1C_ − Ct_u6_) − control group (Ct_SNORD1C_ − Ct_u6_). Specific primers for SNORD1C were purchased from RiboBio (Guangzhou, China).

### 2.3 Cell culture

Normal intestinal epithelial cells NCM460 and human CRC cell lines (SW620, SW480, HCT116, DLD1, HT29, HCT8) were purchased from the Chinese Academy of Sciences Cell Bank (China), and were cultivated in complete Dulbecco’s modified Eagle’s medium (DMEM; Biological Industries, BI) supplemented with 10% fetal bovine serum (Gibco, NY, USA) at 37°C with 5% CO_2_.

### 2.4 Functional enrichment analysis

Co-expressed genes were obtained from TCGA database. GO and KEGG gene functions were enriched using the Database for Annotation, Visualization, and Integrated Discovery (DAVID) (https://david.ncifcrf.gov/) [14,15]. GO analysis included biological process (BP), cellular component (CC), and molecular function (MF) terms. The bubble diagrams of the path process with *P* < 0.01 and the top ten difference were drawn using R language.

### 2.5 Statistical analysis

GraphPad Prism 7.0 and SPSS 22.0 were used for statistical analysis. Mean ± standard deviation (SD) was used to represent the relative expression level of SNORD1C. The expression level of SNORD1C in the serum of different groups showed a skewed distribution. Nonparametric test was used to compare two independent samples and Chi-square test was used for single factor analysis of multiple samples, among which, Kruskal-Wallis test or Mann-Whitey rank sum test were used for comparison between groups. ROC curve was drawn to evaluate the diagnostic performance of SNORD1C. *P* value < 0.05 was considered to be statistically significant.

## 3 Results

In this study, we used qRT-PCR to detect the expression of SNORD1C in the serum of patients with CRC and verified it in the GEO database and in cell lines. The ROC curve was used to evaluate the diagnostic efficacy of SNORD1C expression. The pathological parameters of patients with CRC were used to evaluate the clinical significance of SNORD1C. GO and KEGG enrichment of genes co-expressed with SNORD1C in TCGA may further help to understand the functions and mechanisms of this molecule.

### 3.1 Characteristics of the study participants

**Figure 1.**
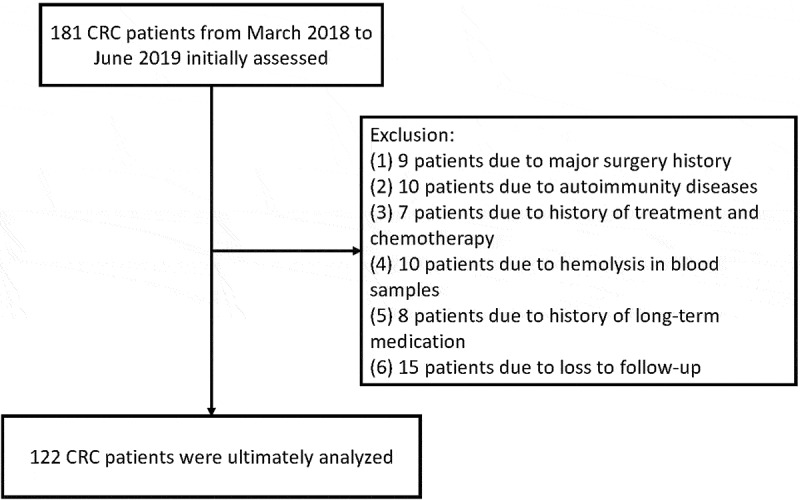
Flow chart showing the screening criteria used for the selection of eligible patients with colorectal cancer. In the course of the study, 181 patients were initially selected and finally 122 (66.9%) met the screening criteria. CRC, colorectal cancer

Initially, 181 patients with CRC were screened. Among them, nine patients had a history of major surgery, ten had autoimmune diseases, seven had a history of chemotherapy, ten had hemolysis in blood samples, eight had a history of long-term medication, and fifteen failed to follow up in time. Ultimately, 122 patients with CRC were selected to participate in the study based on these limitations (Figure 1).

### 3.2 Evaluation of methods for the detection of serum SNORD1C

**Figure 2.**
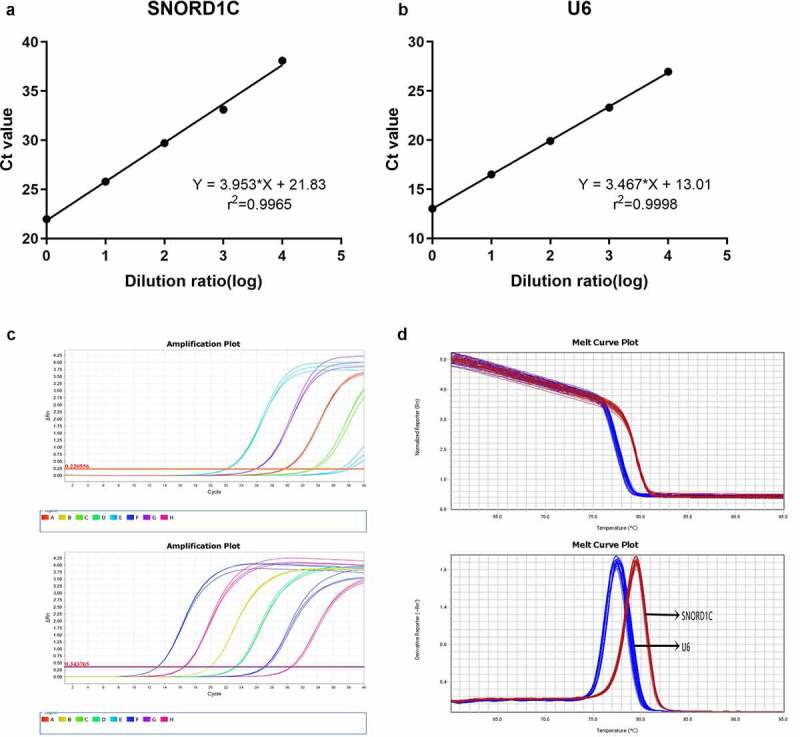
Methodological evaluation of serum SNORD1C. (a-b) The linearity of SNORD1C and U6 are acceptable. (c-d) SNORD1C and U6 melting curve and melting peak showing single peaks and demonstrating good specificity

To evaluate the method of detecting serum SNORD1C, we investigated the stability of reference genes and the linear range, specificity, repeatability, and stability of the assay. To date, there are no universal reference genes for body fluid detection. Therefore, U6, 18S, GAPDH, ACTB, and TUB were selected for qRT-PCR detection in ten mixed serum samples. The results showed that U6 was suitable as the reference gene because of its good linearity and stability (Table 1). Then, to test the range of linearity of SNORD1C, we utilized serial 10-fold dilutions of SNORD1C cDNA to draw the linear regression equation. The r^2^ of the SNORD1C standard curve was 0.9965, and the regression equation was Y = 3.953*X + 21.83. The standard curve of U6 was Y = 3.467*X + 13.01 and r^2^ was 0.9998. These data suggest that qRT-PCR can be used to detect different concentrations of SNORD1C in the serum (Figure 2a-c). The melting curves of both SNORD1C and U6 showed single characteristic peaks, which indicated that the product amplified by this method had high specificity (Figure 2d). Additionally, we conducted intra-assay and inter-assay testing on SNORD1C, and the coefficient of variation was found to be satisfactory (Table 2).

**Table 1.** Ct values of five candidate reference genes

Reference	Mean ± SD	CV, %
U6	22.854 ± 0.045	0.1967
18S	23.127 ± 0.077	0.3346
GAPDH	28.331 ± 0.067	0.2355
ACTB	26.956 ± 0.185	0.6865
TUB	28.186 ± 0.110	0.3919

ACTB, β-actin; GAPDH, glyceraldehyde-3-phosphate dehydrogenase; TUB, tubulin; SD, standard deviation

**Table 2.** Intra-assay coefficient of variation and inter-assay coefficient of variation of SNORD1C

	SNORD1C	U6
Intra – assay		
Mean ± SD	24.621 ± 0.1719	22.261 ± 0.2785
CV, %	0.69	1.25
Inter – assay		
Mean ± SD	23.789 ± 0.6258	22.594 ± 0.4811
CV, %	2.60	2.13

**Figure 3.**
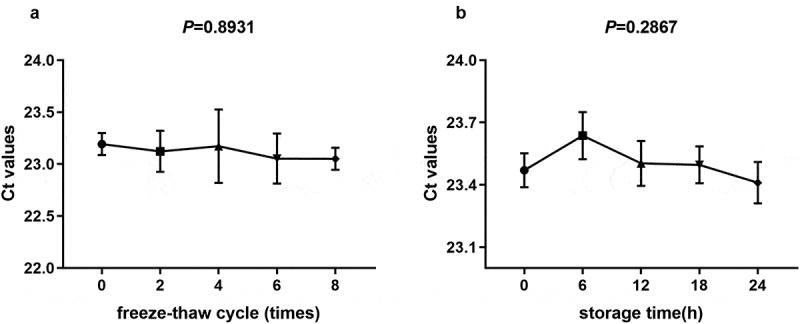
Evaluation of the stability of serum SNORD1C. (a-b) Stability of SNORD1C following repeated freezing and thawing, and at room temperature

After mixing the serum samples at room temperature or following repeatedly freezing and thawing, we analyzed the effects of different standing times (6,12, 18, and 24 h) and freezing and thawing times (0, 2, 4, 6, and 8 times) on the expression of SNORD1C. The results showed that the expression level of SNORD1C was not significantly different (*P* > 0.05, Figure 3a-b). In summary, the qRT-PCR method for detecting SNORD1C has high sensitivity and specificity.

### 3.3 Relationship of serum SNORD1C concentration and CRC

**Figure 4.**
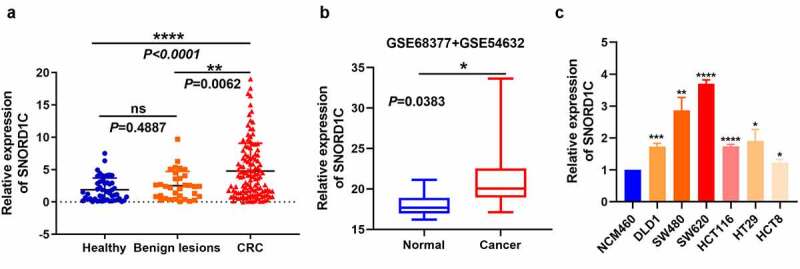
Expression of serum SNORD1C in patients with CRC and GEO database. (a) Scatter plots of serum SNORD1C levels from patients with CRC (n = 122), benign lesions (n = 33), and healthy controls (n = 50). (b) mRNA levels of SNORD1C in CRC and paired adjacent non-cancerous tissues in GSE68377 and GSE54632. (c) Expression of SNORD1C in cell lines. All data are presented as mean ± standard deviation (SD). *P < 0.05; **P < 0.01; ****P < 0.0001

To examine SNORD1C expression level in the serum of different patients, we collected various serum samples. The relative expression of SNORD1C in the serum of 122 newly diagnosed patients with CRC, 33 with intestinal polyps and other lesions, and 50 healthy controls were determined by qRT-PCR, and the data were statistically analyzed using nonparametric tests. Compared to the SNORD1C levels in the serum of healthy individuals (*P* < 0.0001) and those with colorectal benign lesions (*P* = 0.0062), those in the serum of patients with CRC were significantly increased. The expression level of SNORD1C between benign colorectal diseases and healthy subjects was not significantly different (*P* = 0.4887, Figure 4a).

In addition, we analyzed the GSE68377 (n = 7) and GSE54632 (n = 5) databases with non-coding RNA profiling using an array containing 12 pairs of CRCs and para-cancerous tissues. The results showed that SNORD1C expression was higher in the CRC tissues than in para-cancerous tissues, which is consistent with the results of our study (Figure 4b). To verify the high expression of SNORD1C in CRC, we also tested the mRNA expression of SNORD1C in normal intestinal epithelial cells NCM460 and human CRC cell lines, DLD1, SW480, SW620, HCT116, HT29, and HCT8. Consistent with the expression levels in serum and tissues, compared to its expression in NCM460 cells, the expression of SNORD1C was higher in the CRC cell lines, and both the differences were statistically significant (*P* < 0.05, Figure 4c).

The clinicopathological parameters of the 122 patients with CRC were grouped by sex, age, tumor tissue differentiation, depth of invasion, and tumor vascularity and nerve invasion. These grouped clinical data were analyzed using the Chi-square test. High expression of SNORD1C in the serum of patients with CRC was found to correlate with poor tissue differentiation and high CEA levels (*P* < 0.05), whereas it was not significantly associated with the other clinicopathological parameters (*P* > 0.05, Table 3).

**Table 3.** Correlation of SNORD1C expression and clinicopathological characteristics of patients with CRC

Characteristics	n	SNORD1C		p value
		high	low	
Total	122	61	61	
Gender				0.067
Male	89	40	49	
female	33	21	12	
Age (years)				0.716
≤60	66	32	34	
>60	56	29	27	
Location				0.102
Colon	55	23	32	
Rectum	67	38	29	
differentiation				0.928
Well	10	5	5	
moderate	71	37	34	
Poor	14	7	7	
unknown	27	12	15	
Tumor infiltration				0.039*
T1-T2	25	10	15	
T3-T4	54	34	20	
unknown	43	17	26	
Lymph node metastasis				0.374
Yes	35	14	21	
No	65	35	30	
unknown	22	12	10	
Venous invasion				0.644
positive	36	17	19	
negative	61	33	28	
unknown	25	11	14	
Nerve infiltration				0.698
positive	25	14	11	
negative	72	36	36	
unknown	25	11	14	
CEA (ng/ml)				0.034*
≤5	78	37	41	
>5	34	22	12	
unknown	10	2	8	

Note: *p < 0.05

TNM, tumor–lymph node metastasis; CEA, carcinoembryonic antigen

### 3.4 Serum biomarker SNORD1C has good diagnostic value in CRC

**Figure 5.**
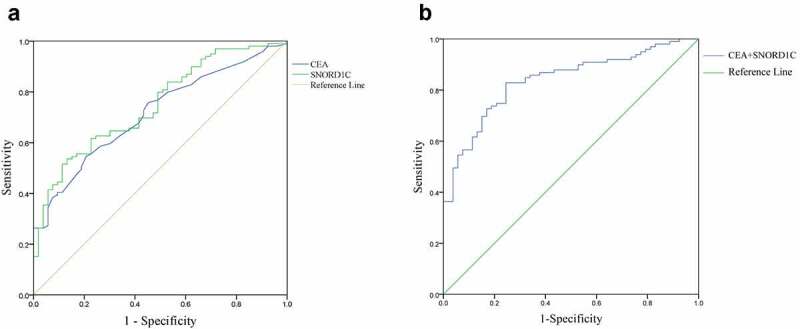
ROC curves to analyze the diagnostic efficacies of SNORD1C and CEA. (a) ROC‐AUC to compare the diagnostic ability of SNORD1C and CEA to discriminate CRC from normal controls. (b) ROC curve of SNORD1C combined with CEA in diagnosis of CRC. ROC, receiver operating characteristic; AUC, area under the ROC curve

To investigate the diagnostic value of SNORD1C, we analyzed 122 patients with CRC and 50 healthy subjects. The ROC curve was used to evaluate the diagnostic efficiency of SNORD1C. ROC curves were drawn based on the expression levels of SNORD1C and CEA in the serum of 99 patients newly diagnosed with CRC and 53 healthy subjects. The results showed that AUC of SNORD1C was 0.748 (sensitivity, 79.80%; specificity, 57.45%; 95% confidence interval [CI], 0.670–0.826; *P* < 0.001) compared to 0.715 for CEA, (sensitivity, 82.83%; specificity, 37.74%; 95% CI: 0.634–0.796; *P* < 0.001), which suggested that SNORD1C may have a higher diagnostic power. These results indicated that SNORD1C could potentially serve as a good biomarker for screening of CRC (Figure 5a). To further evaluate the diagnostic efficacy of SNORD1C, we compared the diagnostic ability of SNORD1C combined with CEA in CRC. The AUC of the combined biomarkers was 0.838 (95% CI: 0.774–0.901; *P* < 0.001), accompanied by a sensitivity of 85.86% and specificity of 62.26%, which was higher than that of the individual biomarkers (Figure 5b).

### 3.5 Preoperative and postoperative differences and prognostic value of serum SNORD1C in CRC

**Figure 6.**
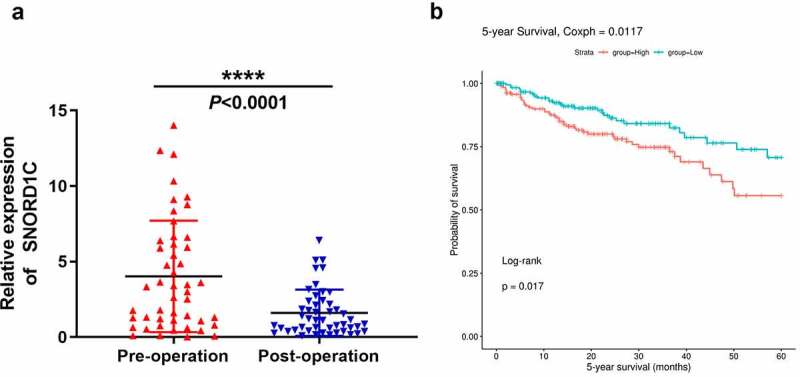
Dynamic monitoring of serum SNORD1C levels and its prognostic value in CRC. (a) Changes of serum SNORD1C levels in 48 newly diagnosed patients with CRC following operation. (b) Overall survival rate of patients with CRC with high vs. low SNORD1C expression levels (http://bioinfo.life.hust.edu.cn/SNORic)

Serum SNORD1C level in 48 newly diagnosed patients with CRC was monitored before and after surgery, and the expression level was analyzed using the Wilcoxon signed-rank test. The SNORD1C level in CRC patients after surgery showed a significant downward trend (*P* < 0.0001, Figure 6a). Moreover, survival analysis of the SNORic database (http://bioinfo.life.hust.edu.cn/SNORic) showed that the five-year survival rate of patients with CRC with high expression of SNORD1C was significantly lower than that of the low expression group (*P* < 0.05, Figure 6b), indicating that SNORD1C is closely related to the prognosis of patients with CRC.

### 3.6 GO and KEGG functional enrichment analysis of SNORD1C in CRC

**Figure 7.**
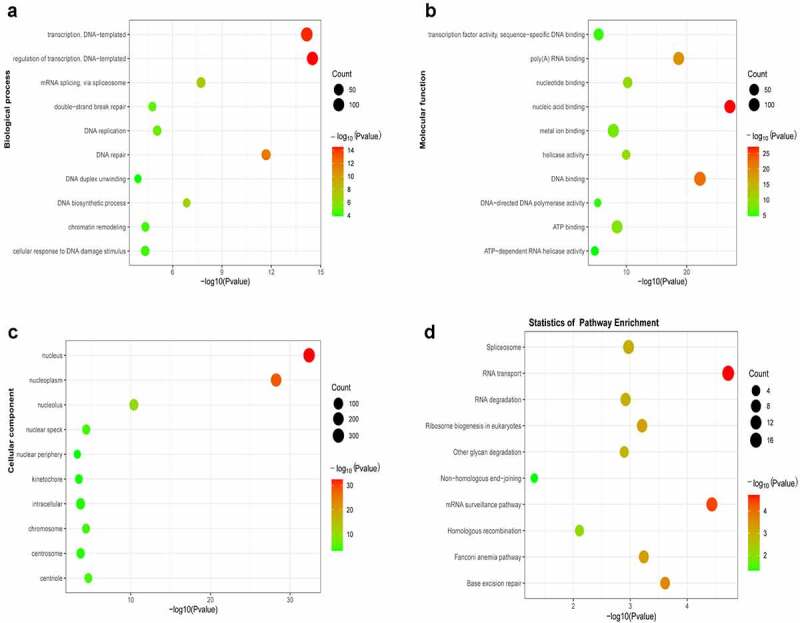
GO and KEGG analysis of co-expressed genes. (a) Top ten biological processes (BPs) of SNORD1C co-expressed genes. (b) Top ten molecular functions (MFs) of SNORD1C co-expressed genes. (c) Top ten cellular components (CCs) of SNORD1C co-expressed genes. (d) KEGG pathway analysis indicating the pathway of SNORD1C co-expressed genes and the top ten pathways. GO, Gene Ontology; KEGG, Kyoto Encyclopedia of Genes and Genomes

The DAVID online tool was used to enrich and analyze the genes co-expressed with SNORD1C in CRC and para-cancerous tissues in the TCGA database. The top ten functional annotations or pathways that differed significantly were identified. GO analysis demonstrated that BPs were mainly involved in DNA transcription, DNA repair, and DNA biosynthesis (Figure 7a). The CCs were mainly involved in the cell nucleus, cell nucleoplasm, and cell nucleolus (Figure 7b). MFs were primarily involved in nucleic acid binding, DNA binding, and poly(A) RNA binding (Figure 7c). Moreover, KEGG pathway enrichment analysis showed that SNORD1C was involved in the RNA transport pathway, mRNA surveillance pathway, ribosome biogenesis in eukaryotes pathway, and spliceosome pathway (Figure 7d). These functions are closely related to the involvement of snoRNAs in the regulation of ribosomes, rRNA processing, RNA splicing, and translation regulation.

## 4 Discussion

According to recent data, CRC ranks second in incidence and third in cancer-related mortality among cancers [16]. Undeniably, the pathogenesis of CRC is complex, and adverse lifestyle, and environmental and genetic factors can all affect the occurrence and development of CRC. Therefore, early cases of CRC in patients are often missed or misdiagnosed because of atypical clinical symptoms. Traditional CRC screening includes fecal occult blood tests, colonoscopy, and blood CEA detection [17]. However, due to low patient compliance and the low sensitivity of these methods, early diagnosis of CRC remains difficult. The emergence of liquid biopsy technology has brought new hope for the early diagnosis of diseases [18]. Nucleic acids, peptides, proteins, and intact tumor cells in body fluids can be detected by this method. Minimally invasive methods such as the liquid biopsy technique are preferred for the diagnosis and evaluation of patient outcomes, which is immense value for improving the clinical management of cancer patients [19,20]. Hence, finding new biomarkers to supplement CRC screening and diagnostic methods is of vital clinical value.

SnoRNAs are short ncRNAs that guide the post-transcriptional modification of ribosomes and small nuclear RNA (snRNA) [21]. Most of the host genes of snoRNAs encode proteins or transcripts that are necessary for ribosome biosynthesis and function. Several studies have found that snoRNAs trigger a series of carcinogenic effects through their own abnormal expression, thereby affecting the occurrence of most tumors, including CRC [22]. Su et al. [23] found two snoRNAs, SNORD66 and SNORD78 in sputum samples, which can be used as biomarkers for the diagnosis of lung cancer. Studies have also shown overexpression of SNORD78 in non-small cell lung cancer [24]. In addition, a study reported that abnormally high expression of SNORA55 promotes proliferation and migration of prostate cancer cell lines [25]. In their study, Li et al. [26] reported that the signal transduction mechanism mediated by snoRNAs may promote the occurrence and development of gastric cancer. Yang et al. [27] emphasized that during the process of evolution of chronic enteritis into malignant tumors, three snoRNAs, including SNORD33, SNORA15, and SNORA41, may be involved in carcinogenesis and promote its progression. Zhang et al. [28] used qRT-PCR to compare dozens of pairs of CRC tissues and normal colon tissues, and found that the abnormal expression of SNORA71A was significantly related to TNM staging and lymph node metastasis, and can be used as a biomarker to assist in the diagnosis of CRC. Similarly, in CRC tissues, SNORA21 was shown to have significant value in the prognosis of patients and was highly correlated with distant metastasis of CRC [8]. The growing role of snoRNAs in cancer research has been demonstrated in several studies, which should not be ignored.

However, there are very few studies on serum snoRNAs as biomarkers of CRC. In non-small cell lung cancer, SNORD1C expression was more likely to be expressed in ALDH1+ tumor cells, and we suspected that SNORD1C may be a tumorigenic marker [11]. In our study, we identified SNORD1C by screening the GEO and TCGA databases at an early stage of CRC. We first evaluated the performance of serum SNORD1C in CRC by qRT-PCR and found that the specificity and repeatability of SNORD1C in qRT-PCR were satisfactory. Therefore, we hypothesized that serum snoRNA has the potential to serve as a noninvasive biomarker. Compared to the benign colorectal disease group and the healthy (physical examination) group, the expression level of serum SNORD1C in patients newly diagnosed with CRC was significantly higher. Analyses of the GSE68377 (n = 7) and GSE54632 (n = 5) datasets revealed that the expression of SNORD1C was higher in CRC tissues than that in para-cancerous tissues. We also found augmented levels of SNORD1C in human CRC cell lines, compared to NCM460. To further study the clinical value of serum SNORD1C, we compared the AUC of SNORD1C and CEA. Interestingly, we found that the AUC of SNORD1C was higher than that of CEA, which is advantageous for the diagnosis of CRC. Moreover, survival analysis in the SNORic database showed that higher expression of SNORD1C was related to poor prognosis of CRC. We then performed GO and KEGG analysis on the co-expressed genes and found that the enrichment pathway processes were associated with the general functions of the ribosomal modification of snoRNA, RNA transduction, and DNA repair.

These results indicate that serum SNORD1C may be a potential marker for the diagnosis and prognosis of CRC. However, a specific limitation of this study was that the sample size examined was relatively small, and more samples need to be analyzed to confirm our conclusions. Furthermore, the underlying mechanism of SNORD1C in CRC remains to be clarified, and we will explore this in the future to further elucidate the potential of SNORD1C in CRC treatment.

## 5 Conclusion

In summary, the diagnostic role of serum SNORD1C and enrichment analyses of SNORD1C in CRC were conducted in this study. We identified a key role of SNORD1C in CRC. The high expression of SNORD1C was related to poor differentiation of CRC tissues and high CEA levels. Our research indicated that serum SNORD1C may be used as a new biomarker for the clinical diagnosis and prognostic evaluation of CRC.

## Data Availability

Data supporting the findings of this study are available from the corresponding authors on reasonable demand. The authors acknowledge the TCGA database (https://tcga-data.nci.nih.gov/tcga), the GSE68377 and GSE54632 datasets in the GEO database, and the SNORic database (http://bioinfo.life.hust.edu.cn/SNORic) for their contributions.

## Highlights

1. Serum SNORD1C was upregulated in CRC and associated with poor prognosis.
2. The AUC of combined SNORD1C and CEA levels was higher than that of the individual parameters.
3. High SNORD1C in CRC was related to poor tissue differentiation and high CEA level.
4. GO and KEGG analysis were performed on SNORD1C co-expressed genes in TCGA.
